# OrchidMD: An Integrated and User‐Interactive Orchid Multi‐Omics Database for Mining Genes and Biological Research

**DOI:** 10.1111/pbi.70445

**Published:** 2025-11-11

**Authors:** Yonglu Wei, Zengyu Lin, Qi Xie, Jie Gao, Jianpeng Jin, Jie Li, Chuqiao Lu, Guangying Ye, Wenkang Li, Chuanfeng Huang, Dengqi Yang, Qi Liu, Genfa Zhu, Fengxi Yang

**Affiliations:** ^1^ Guangdong Key Laboratory of Ornamental Plant Germplasm Innovation and Utilization, Environmental Horticulture Research Institute, Guangdong Academy of Agricultural Sciences Guangzhou China; ^2^ Department of Mathematics and Computer Science Dali University Dali Yunnan China; ^3^ Rice Research Institute, Guangdong Academy of Agricultural Sciences, Guangdong Key Laboratory of Rice Science and Technology, Guangdong Rice Engineering Laboratory, Key Laboratory of Genetics and Breeding of High Quality Rice in Southern China (Co‐Construction by Ministry and Province) Guangzhou China

**Keywords:** ARF, database, functional genomics, multi‐omics, Orchidaceae

## Abstract

The Orchidaceae family, with its unparalleled species diversity among angiosperms, is integral to ornamental, medicinal, cultural, and ecological value. Multi‐omics techniques have proven invaluable for the identification of candidate genes and the advancement of functional genomics research. Nevertheless, the application of these technologies in Orchidaceae remains severely limited due to the lack of effective platforms that can integrate and analyze multi‐omics data, especially in understanding the mechanisms underlying key traits such as distinctive floral morphology. In this study, we present OrchidMD, the Orchid Multi‐omics Database (www.orchidcomics.com), a resource platform that integrates data from five omics layers: genomics, transcriptomics, proteomics, metabolomics, and phenomics, encompassing a total of 213 species. OrchidMD is equipped with 18 specialized statistical and analytical tools, and features a user‐friendly interface that facilitates efficient gene mining, multi‐omics data exploration, and integrative interactive analysis. A case study on the comprehensive identification of the pan‐ARF gene family across Orchidaceae species demonstrates the effectiveness and convenience of OrchidMD. Furthermore, experimental validation further shows that transgenic overexpression of *CsiARF04* promotes the differentiation and budding of orchid rhizomes. In addition, another case study using gene editing in orchids, CRISPR Design was employed to predict the *CsiPDS* target site in *Cymbidium sinense*. Effective editing was subsequently achieved via *Agrobacterium*‐mediated delivery of the CRISPR/Cas9 vector into leaves. These results underscore OrchidMD‘s formidable capacity to discern candidate genes associated with salient traits and elucidate their regulatory mechanisms. Thus, OrchidMD serves as a pivotal platform advancing multi‐dimensional biological research and functional genomics in orchids.

## 1 Introduction

The Orchidaceae family, one of the largest and most ecologically important groups of flowering plants (Givnish et al. 2015; Zhang et al. 2023), has attracted substantial attention due to its ornamental, medicinal, ecological, and cultural importance (Zhang et al. 2022; Zimmerman and Whigham 1992). Orchids possess remarkable adaptability, thriving in diverse environments, from tropical rainforests to arid deserts. These adaptations are often linked to their unique reproductive strategies and specialised floral structures (Li et al. 2022). Moreover, the fungus‐specific mycorrhizal symbiosis and unique seed‐germination mechanisms (Zhao et al. 2024), the evolution of crassulacean acid metabolism (CAM) photosynthesis closely associated with the epiphytic lifestyle (Gamisch et al. 2021), slow reproductive development (Jersáková et al. 2006), large and complex genomes (Trávníček et al. 2019; Yang et al. 2025), and unconventional vegetative propagation strategies (Lee 2018) further underscore the distinctive biological features of orchids. These characteristics pose significant challenges for omics‐based research, yet at the same time they provide unique opportunities to uncover novel regulatory mechanisms and gain deeper insights into plant evolutionary processes.

In order to achieve a more profound comprehension of the molecular mechanisms that underpin orchid trait development, it is imperative to delve into the intricacies of the genome, transcriptome, proteome, metabolome and phenome (Chen, Wang, et al. 2024; Freudenstein 2024; Li et al. 2022; Tiwari and Chen 2023). In recent years, multi‐omics approaches have greatly expanded our capacity to reveal the complex biological processes that govern these traits (Ahmad et al. 2024; Wen et al. 2024). By leveraging multi‐omics datasets, researchers have developed sophisticated methodologies for identifying genetic loci and candidate genes that regulate key orchid phenotypes. Techniques such as genome‐wide association studies (GWAS) (Yang, Guo, Li, et al. 2023) and expression quantitative trait locus (eQTL) mapping (Li et al. 2019) have successfully pinpointed genetic factors influencing flower morphology, pollination strategies, and other critical traits. Consequently, these studies provide valuable insights for orchid breeding and conservation efforts (Jiang et al. 2024; Russo et al. 2024). These tools are increasingly vital for the breeding of orchids and other crops (Sedeek et al. 2023).

Building on these advances, the establishment of multi‐omics platforms in major crops such as rice ( *Oryza sativa* ) (Sakai et al. 2013; Yu et al. 2023), wheat ( *Triticum aestivum* ) (Ma et al. 2021), maize ( *Zea mays* ) (Han et al. 2023), soybean ( *Glycine max* ) (Liu et al. 2023; Yang et al. 2024), and cotton (Yang, Wang, Huang, et al. 2023), as well as in horticultural species including oilseed rape (Yang, Wang, Wei, et al. 2023), citrus (Liu, Wang, et al. 2022), and bamboos (Bambusoideae, Poaceae) (Liu et al. 2024), researchers have gained the ability to analyse a wide range of genomic, transcriptomic, metabolomic, and proteomic data, significantly advancing our understanding of these species (Mansoor et al. 2024; Perez‐Riverol et al. 2017). Orchids present unique challenges in omics research due to their specialised floral structures and complex genomic architecture (Jiang et al. 2024; Li et al. 2025). Therefore, there is a critical need for a specialised platform that systematically integrates multi‐omics data to support functional genomics research and breeding applications (Balilashaki et al. 2023).

Since the release of OrchidBase in 2011, which included 11 in‐house Phalaenopsis orchid cDNA libraries (Fu et al. 2011), an increasing number of sequencing datasets have been generated and made publicly available. Notably, in recent years, multi‐omics technologies have significantly advanced the identification of key candidate genes and loci associated with important orchid traits, including floral patterning, colour, fragrance and floral development (Hsu et al. 2015; Liu, Leng, et al. 2022). These advances have been supported by databases like Orchidstra 2.0 (Chao et al. 2017) and OrchidBase 6.0 (Chen, Sun, et al. 2024), which provide valuable reference data to further enhance multi‐omics research. Nevertheless, existing databases have struggled to integrate and interactively manage multi‐omics data for orchids, presenting significant challenges in handling the growing volume of such data (Zhang et al. 2023). To address this critical gap, we have developed the Orchidaceae Multi‐omics Database (OrchidMD) (http://www.orchidcomics.com), an integrative and user‐friendly platform designed to streamline the storage, visualisation, and analysis of orchid omics data. OrchidMD consolidates high‐quality genomic assemblies, resequencing datasets, and phenotypic records for representative orchid germplasms (Cai et al. 2015; Yang, Guo, Li, et al. 2023; Zhang et al. 2017), alongside transcriptomic, proteomic, and metabolomic profiles collected from diverse tissues, organs, and accessions (Ahmad et al. 2024; Fan et al. 2023). Furthermore, it incorporates results from QTL mapping and GWAS to support genetic dissection of complex traits. In addition to conventional analysis modules such as gene annotation, expression exploration, and network construction, OrchidMD features a built‐in CRISPR/Cas target site design tool tailored to orchid genomes. While numerous online platforms, including CRISPR‐GE (Xie et al. 2017), support gene editing in model species, orchid researchers have long lacked a dedicated system for efficient and accurate target design. By integrating this functionality within OrchidMD, we aim to bridge the gap between multi‐omics insights and practical genome engineering, enabling functional validation and accelerating molecular breeding in orchids. Together, these capabilities position OrchidMD as a powerful resource for the orchid research community worldwide.

## 2 Results

### 2.1 Content and Overview of OrchidMD

To provide a comprehensive view of orchid multi‐omics data, we integrated original datasets across five omics layers from recent studies and existing resources. These datasets include genomes of 22 orchid species, transcriptomes of 767 libraries from 174 species and/or cultivated varieties (Table S1), phenotypes of 227 varieties (Table S2) as well as the proteomes (Table S3) and metabolites across 51 orchid accessions (Table S4). Additionally, a genome‐wide association study (GWAS) dataset has been included, featuring 65 318 522 single nucleotide polymorphisms (SNPs) and 3 906 176 structural variants (SVs).

**FIGURE 1:**
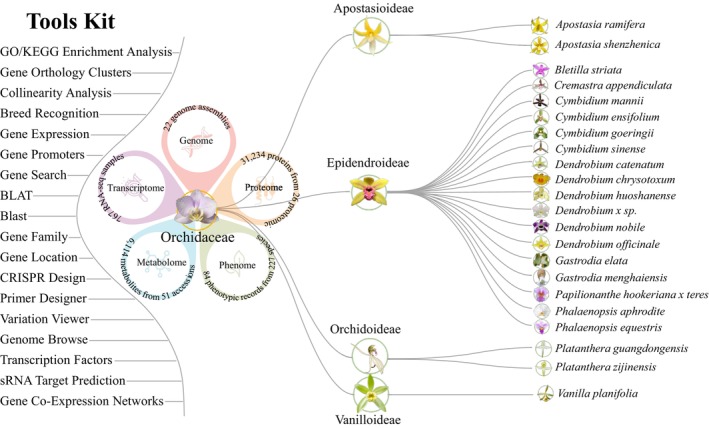
Schematic design and functions in Orchidaceae multi‐omics analysis.

The integration and analysis of these diverse datasets culminated in the creation of the Orchid Multi‐omics Database (OrchidMD; https://www.orchidcomics.com), which is illustrated in Figure 1. OrchidMD features 23 distinct modules, including Genome Browse, Gene Search, Gene Expression, GO/KEGG Enrichment Analysis, Transcription Factors, Breeding Recognition, Gene Orthology Clusters, Gene Promoters, BLAST, BLAT, GWAS, Gene Family, Primer Designer, sRNA Target Prediction, Gene Co‐Expression Networks (GCNs), Gene Location, Collinearity Analysis, CRISPR Design, Transcriptome, Phenotype, Metabolome, Proteome, and Variation Viewer. The database holds a total of 329.40 Gb of data, which is curated using a combination of structural and homology‐based multi‐omics methods, along with network tools for analysis. OrchidMD provides a wide array of visual tools to facilitate browsing and comparison of resource available information, including genome sequences, gene structures, metabolite contents, and phenotypic data. These features are designed to streamline resource management and aid researchers in exploring the regulatory mechanisms and evolutionary pathways underlying gene function in orchids.

### 2.2 Features in Omics Portals

#### 2.2.1 Genomics

**FIGURE 2:**
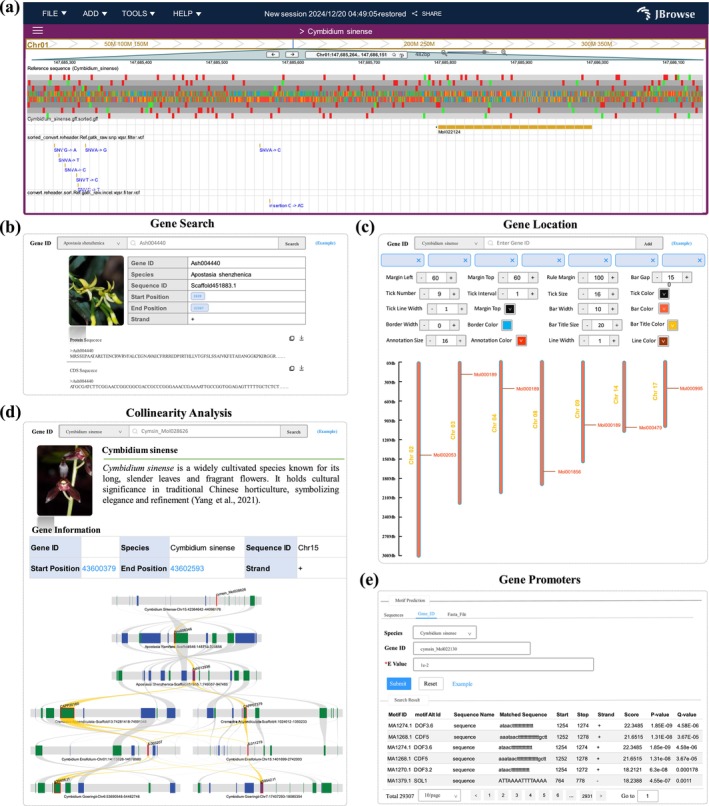
Sequence analysis modules in OrchidMD. (a) Web interface with graphical visualisations, including JBrowse genome browser for SNP information. (b) Gene search. (c) Gene location. (d) Collinearity analysis. (e) Gene promoters.

OrchidMD encompasses a comprehensive repertoire of 22 high‐quality genome assemblies representing orchid species, comprising 17 from the subfamily Epidendroideae, two from Apostasioideae, two from Orchidoideae, one from Vanilloideae (Figure 1). To facilitate genome exploration, users can access the Browse module (Figure 2a) to input gene identifiers or chromosomal intervals, enabling the visualisation of gene structures, variation sites, genome sequences, and annotations. Corresponding sequence data and annotations are available for download through the Download module. In addition, the Gene Search module (Figure 2b) allows users to retrieve specific gene and protein sequences by gene ID. The Gene Location module (Figure 2c) further enables users to generate personalised chromosome location maps by simply providing the gene ID. Beyond basic access, OrchidMD also supports a wide range of comparative genomic analyses. For instance, the platform has mapped 1 484 675 interaction relationships among 395 908 proteins and identified 123 038 synteny blocks across all included Orchidaceae species. The Collinearity Analysis allows users to rapidly investigate the evolution and diversification of large syntenic gene blocks. By entering a gene ID, users can automatically generate and save vector graphics for further analysis (Figure 2d), thereby supporting studies of gene gain or loss.

Moreover, all promoter regions have been incorporated into the database, and users can easily identify motifs within these regions by inputting gene IDs or uploading custom sequences through the Gene Promoters module (Figure 2e). OrchidMD also includes 35 122 transcription factors (TFs), which can be browsed using the Transcription Factors module in the Tools portal. Furthermore, users can perform sequence searches using the BLAT and Blast tools. For gene family comparative genomics, OrchidMD integrates OrthoVenn3 (Sun et al. 2023) via the Gene Orthology Clusters modules. This interactive tool allows users to explore homologous gene groups and species‐specific gene presence or absence events.

In total, OrchidMD houses data on 569 981 genes, with functional annotations available for 432 716 of these genes. All genome synteny and gene indexing have been updated and can be accessed via the GBrowser and Collinearity Analysis modules. The Gene Search module is designed for users to browse and download gene sequences directly.

#### 2.2.2 Transcriptomics

**FIGURE 3:**
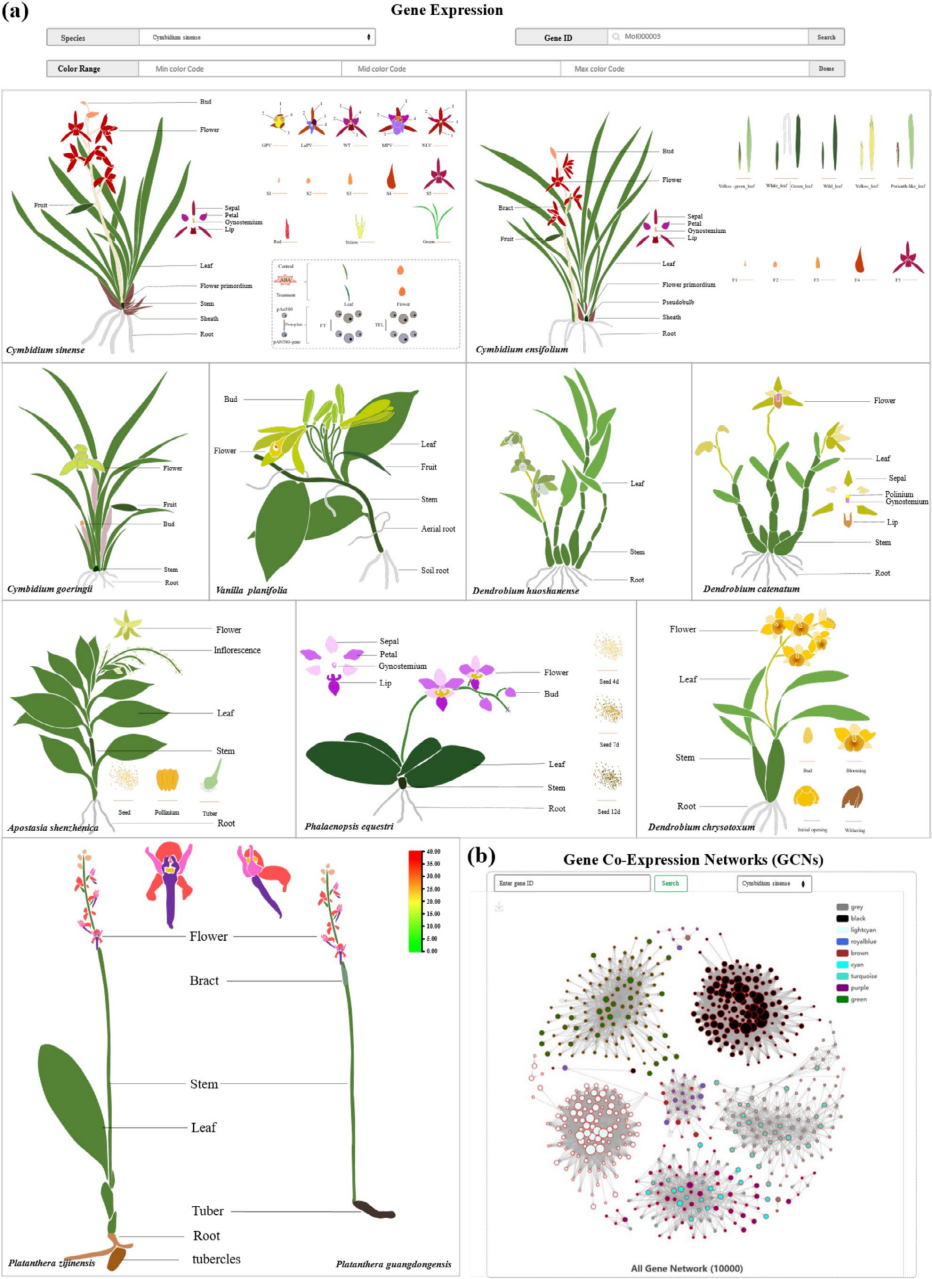
Expression data exploration modules in OrchidMD. (a) Cartoon heatmaps available on OrchidMD for *Cymbidium* sinense, *Cymbidium* ensifolium, *Cymbidium* goeringii, *Vanilla planifolia* , Dendrobium huoshanense, Dendrobium catenatum, Apostasia shenzhenica, Phalaenopsis equestris, Dendrobium chrysotoxum, Platanthera zijinensis and Platanthera guangdongensis. (b) Gene Co‐Expression Networks (GCNs).

For the transcriptomics portal, we curated 767 RNA sequencing (RNA‐seq) libraries representing diverse experimental conditions, including multiple tissues, hormone and stress treatments, photoperiod regimes and circadian conditions, developmental stages, and symbiotic or non‐symbiotic states. These datasets span 93 BioProjects, 614 BioSamples, and 174 species or cultivars (Table S1). Importantly, gene expression is pivotal for the synthesis of functional gene products that influence phenotypic traits. OrchidMD's Gene Expression portal provides multiple interfaces for exploring expression data (Figure 3). Notably, the spatiotemporal expression visualisation using the electronic Fluorescent Pictograph (eFP) system, allows for intuitive visualisation of gene expression patterns across various orchid species (Figure 3a). eFP profiles have been created for all 11 plants in the database, enabling users to analyse gene expression distribution by entering gene identifiers.

In particular, OrchidMD includes RNA sequencing data from 225 RNA‐seq libraries, providing a comprehensive one‐stop solution that enables users to visualize, analyze, and interpret spatiotemporal gene expression data. It covers 16 distinct tissues and organs, including seed, soil root, aerial root, stem, leaf, flower, inflorescence, fruit, lateral sepal, dorsal sepal, sepal, lip, column, pollinia, ovary, and bract. Specifically, the dataset encompasses 3 seed development stages (Seed 4d, Seed 7d and Seed 12d), 5 typical flower type categories (wild type (WT), labellum‐like perianth variety (LaPV), multi‐perianth variety (MPV), null‐lip variety (NLV) and Genostemium‐like perianth variety (GPV)), 6 different flower development stages (floral primordium, 1–5 mm floral bud, 6–10 mm floral bud, 11–15 mm floral bud, 16–20 mm floral bud, fade flower), 7 leaf variegation types (wild leaf, yellow‐green leaf, yellow leaf, red leaf, white leaf and perianth‐like leaf) as well as hormone treatments. All data have been collected, processed, and summarized, facilitating the quick inspection of gene expression patterns across various experiments or conditions. Furthermore, OrchidMD integrates gene co‐expression data, constructing comprehensive Gene Co‐Expression Networks (GCNs); in total, 8 881 927 co‐expression gene pairs have been identified, enabling users to identify genes co‐expressed with genes of interest by entering the gene identifier (Figure 3b).

#### 2.2.3 Phenome

**FIGURE 4:**
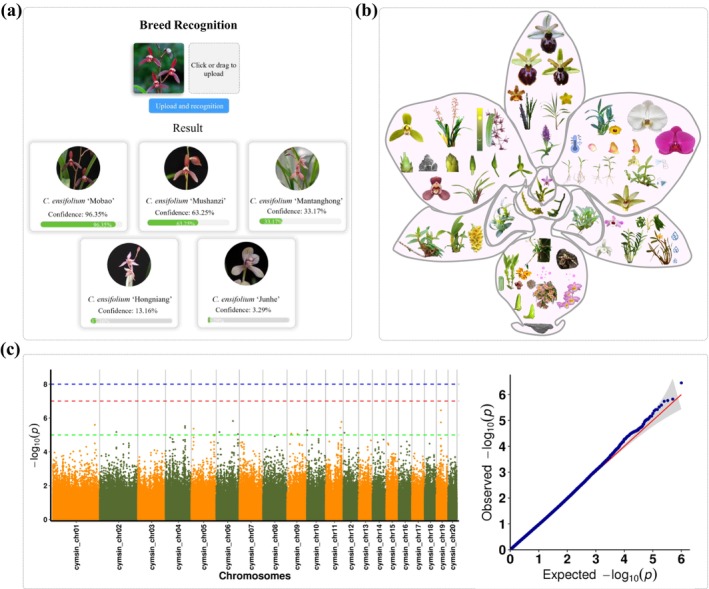
Multi‐omics data and applications in OrchidMD. (a) Example of the Breed Recognition module for orchid species identification. (b) Species and tissue types included in the collected proteomic and transcriptomic datasets. (c) Representative results from genome‐wide association studies (GWAS) conducted within OrchidMD.

Orchid species identification, particularly within the *Cymbidium* genus, presents significant challenges due to limited classification methods, a lack of expert knowledge, and weak conservation awareness. To address these challenges, OrchidMD integrates 69 224 698 genetic variations and 84 phenotypic traits from 227 core *C. sinense* accessions (Yang, Guo, Li, et al. 2023). In addition, we have also incorporated 156 630 images of *Cymbidium* species to enhance species identification. To improve the accuracy, we developed a deep convolutional neural network (CNN) enhanced with squeeze‐and‐excitation (SE) blocks and residual connections. SE blocks help highlight critical features, while residual connections ensure efficient gradient flow. Hyperparameter tuning and Principal Component Analysis (PCA) were applied to optimize the model's performance. The model achieved an impressive initial training accuracy of 99.21% and a validation accuracy of 86.50%. These results demonstrate the efficacy of our approach, offering a promising solution for the automated identification of *Cymbidium* species. Moreover, users can upload photos of orchid species to the Breed Recognition module within OrchidMD, where the system automatically analyzes the images and returns the closest matching species, facilitating rapid and accurate species identification (Figure 4a).

#### 2.2.4 Proteome and Metabolome

The Proteome and Metabolome modules of OrchidMD integrate diverse datasets spanning multiple orchid species, developmental stages, environmental conditions, and cultivation practices, thereby providing a valuable resource for functional and comparative studies (Figure 4b), Within these modules, the numerical values displayed for each proteomic or metabolomic dataset are interactive; by clicking on a number, users are directed to the corresponding detailed listing page.

In the Proteome portal, OrchidMD incorporates 26 proteomic datasets covering various orchid species and experimental conditions. Key datasets involve the proteomic analysis of *Cymbidium sinense* across six floral developmental stages and between green and yellow‐leafed mutants, *Cymbidium goeringii* floral organs (sepals, petals, lip, and column), *Paphiopedilum barbigerum* seeds inoculated with *Epulorhiza* sp. FQXY019, *Dactylorhiza majalis* tubers under sterile and symbiotic fungal culture conditions, *Ophrys* species (ex. *Ophrys exaltata*, *Ophrys sphegodes*, *Ophrys garganica*) flowers, and *Dendrobium huoshanense* under lead and salt stress, as well as whole‐plant profiling. Additionally, *Phalaenopsis amabilis* mixed floral buds were analysed under different temperature treatments. Together, these datasets offer comprehensive insights into protein expression related to orchid development, stress responses, and symbiotic interactions.

The Metabolome Portal, OrchidMD integrates 51 high‐resolution metabolomic datasets to systematically characterize metabolic variations across 18 orchid species, including developmental stages, environmental conditions, and cultivation methods, with key datasets including organ‐specific metabolic profiling of roots, pseudobulbs, tubers, rhizomes, leaves, flowers. In particular, *Cymbidium* species featuring rhizome pigmentation (green vs. yellow cultivars), foliar chromatic variation (red, green, and yellow leaves), six‐stage floral developmental trajectories, and seven time‐point circadian metabolic dynamics under 12 h photoperiod, as well as *Dendrobium* investigations encompassing whole‐plant metabolomes of two species, flower colour differentiation (purple vs. white cultivars), scent compound biosynthesis (high‐scented vs. light‐scented varieties), ontogenetic metabolic shifts (juvenile vs. mature stages), cultivation‐mediated responses across three substrates (arboreal, sandstone, gravel) and growth systems (epiphytic vs. greenhouse conditions), plus humidity gradient acclimation (≥ 95% to ≤ 5% RH), complemented by flower colour differentiation and organ‐specific metabolism (leaves, internodes, aerial roots) of other orchid species. Collectively establishing an unprecedented multidimensional resource for decoding orchid‐specific metabolic pathways, elucidating environmental adaptation mechanisms, identifying species‐specific biochemical signatures, and informing precision cultivation protocols in Orchidaceae research and biotechnology applications.

#### 2.2.5 Integration of GWAS With of Multi‐Omics Data

The present study was conducted with the objective of investigating the association of genetic variations and candidate genes with specific phenotypes. To this end, a comprehensive set of population‐level multi‐omics data was collected and subsequently analyzed using GWAS. By using the Variation Viewer module, users can quickly locate the variations of a specific gene or a specific location (Figure 4c). Furthermore, in order to provide a more convenient and user‐friendly platform for researchers, some common bioinformatics analysis tools were developed and integrated, including GO and KEGG enrichment analysis, primer design and sRNA target prediction. These tools were integrated into the tools portal. It is noteworthy that the majority of analysis tools provide support for 22 published Orchid genomes, thereby enabling users to conduct these analyses without the necessity of switching between different platforms.

### 2.3 Case Study 1: Characterisation of a Transcription Factor Related to Auxin Regulates

**FIGURE 5:**
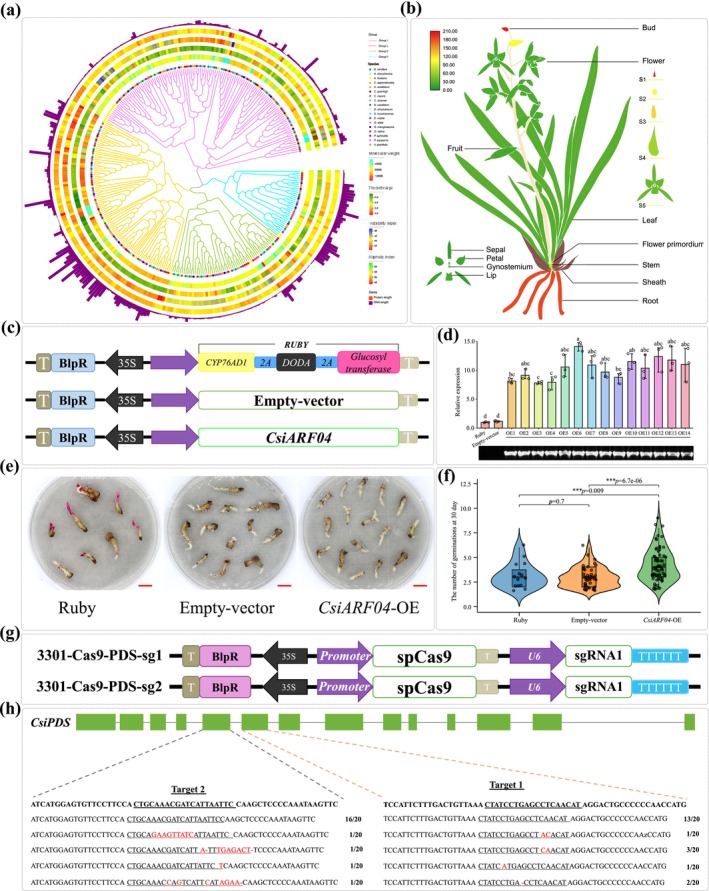
Identification of the ARF gene family in OrchidMD. (a) Phylogenetic tree of ARF genes in *Apostasia ramifera*, *Apostasia shenzhenica*, *Apostasia shenzhenica*, *Cremastra appendiculata*, *Cymbidium ensifolium*, *Cymbidium goeringii*, *Cymbidium mannii*, *Cymbidium sinense*, *Dendrobium catenatum*, *Dendrobium chrysotoxum*, *Dendrobium huoshanense*, *Dendrobium nobile*, *Gastrodia elata*, *Gastrodia menghaiensis*, *Oryza sativa* , *Phalaenopsis aphrodite*, *Phalaenopsis equestris* and *Vanilla planifolia* . (b) Expression profiles of *CsiARF04* across RNA‐seq experiments. (c) Schematic diagram of vector constructs: Marker gene (3302‐Ruby), empty vector control (3302‐EV), and target gene construct (3302‐*CsiARF04*). (d) Expression analysis of *CsiARF04* genes in rhizomes by qRT‐PCR. ata were analysed using one‐way ANOVA followed by Tukey's HSD test; different lowercase letters indicate statistically significant differences (*p* < 0.05) among groups. Error bars represent ± SD. The electrophoresis image below confirms DNA‐level amplification and the identification of transformed rhizomes overexpressing *CsiARF04*. Ruby and Empty‐vector were used as controls. (e) Rhizome phenotypes of *Cymbidium* after overexpression of the *CsiARF04* gene, including plants transformed with the marker gene (Ruby), empty vector control, and target gene (*CsiARF04*‐OE), with a scale bar of 1 cm. (f) Quantification of newly emerged bud numbers in *Cymbidium* rhizomes 30 days after transformation in plants carrying the marker gene (Ruby), empty vector, or target gene (*CsiARF04*‐OE). Data are presented as mean ± SD. Statistical analysis was performed using one‐way ANOVA. *p* value: ***p < 0.001. (g) Schematic diagram of the gene editing vector. The vector's sgRNA comprises a target site and gRNA scaffold. Target site sequence for sgRNA1 (Target 1): CTATCCTGAGCCTCAACAT, target site sequence for sgRNA2 (Target 2): GAATTAATGATCGTTTGCAG. U6 in purple frame represent endogenous U6 promoter of *C. sinense* , TTTTT in a blue box represent the U6 terminator. (h) Analysis of the *CsiPDS* gene structure and editing sites. Green box means the exon sequences of *CsiPDS*. Target sites are located in the fifth exon (Target 2) and sixth exon (Target 1). wild‐type sequence are marked with bold black, target sites are marked with underlined sequences, mutation are marked with red. Numerical annotation: Mutation type (number of mutated clones/total clones).

To demonstrate the platform's capabilities, we present a case study focusing on the analysis of a gene family involved in the regulation of diverse morphogenetic processes, including flowering and seed development, which are key developmental traits in orchids. The pan‐Auxin Response Factor (pan‐ARF) family, characterised by the conserved Auxin_resp domain (PF06507) responsible for transcriptional regulation, plays a central role in mediating auxin signalling pathways during plant development (Hernández‐García et al. 2024); however, their functional roles in orchids remain largely uncharacterized. To address this, we utilised OrchidMD to retrieve genes containing the conserved ARF domain through the Transcription Factors and Gene Family modules. Using this approach, we identified 281 pan‐ARF transcription factors (TFs) from 16 orchid species, which were subsequently classified into four subfamilies (Figure 5a, Table S5). Based on expression pattern and co‐expression analysis, we found *CsiARF03* and *CsiARF04* closely related to flower development, *CsiARF03* (*Mol004175*), whose promoter region contains a CArG‐box DNA binding motif. As a downstream gene of *APETALA1* (*AP1*), *CsiARF03* potentially regulates flower development and inflorescence architecture (Ahmad et al. 2024). Importantly, we further investigated the function of *CsiARF04* (*Mol004325*) utilising the toolkits of OrchidMD. For the expression profile of *CsiARF04*, we utilised OrchidMD's spatiotemporal gene expression visualisation tool, which generated heatmaps indicating that *CsiARF04* is predominantly expressed during floral bud and root development (Figure 5b). To explore the potential function of *CsiARF04* in orchids, we overexpressed *CsiARF04* in the rhizomes of *C. sinense* using the CaMV 35S promoter. A marker gene (3302‐Ruby) and an empty vector (3302‐EV) were used as controls (Figure 5c). Transgenic plants exhibited significantly accelerated rhizome differentiation and budding, exhibiting first bud emergence 10.34 days (59.56%) earlier than the control (7.02 ± 0.82 days versus 17.36 ± 1.28 days) and 9.76 days (58.16%) earlier than the control (versus 16.78 ± 1.72 days), with all comparisons showing statistical significance (*p* < 0.001). At 30 days post‐transformation, the transgenic lines produced a higher median number of buds (4.00, IQR 3.00–5.00) compared to both 3302‐Ruby (3.00, IQR 2.00–3.00) and 3302‐EV controls (3.00, IQR 2.00–4.00), as shown in Figure 5d–f.

### 2.4 Case Study 2: Effective Editing Sites of *CsiPDS* Were Predicted by CRISPR Design

Using the CRISPR Design tool within the OrchidMD platform, we predicted target sites in the *phytoene desaturase* gene of *C. sinense* (*CsiPDS*). Based on these predictions, we constructed two gene‐editing vectors targeting their respective sites (Figure 5g). Each vector was built on the pCAMBIA3301 backbone and integrated an spCas9 expression cassette along with a U6 promoter‐driven sgRNA expression cassette. Subsequently, *C. sinense* leaves were agroinfiltrated using these vectors. To assess editing efficiency, target gene loci were amplified by PCR. The purified PCR products were ligated into a sequencing vector, and 20 single clones were subsequently picked for sequencing analysis. The results revealed that for Target site 1, four distinct mutation types were identified in 35% of the clones sequenced, primarily involving single‐nucleotide edits. Similarly, Target site 2 showed four distinct mutation types in 20% of clones, including three mutants exhibiting multi‐nucleotide deletions (Figure 5h).

## 3 Discussion

In this study, multi‐omics data from recent research and existing databases were collected and processed to construct OrchidMD, the first comprehensive multi‐omics database dedicated to orchids. OrchidMD serves as a powerful resource for researchers, providing genome sequences, annotations, gene expression levels, metabolite contents, phenotypic data, and associated genetic variation signals. Furthermore, OrchidMD integrates a range of online tools for analysis and visualisation, empowering users to explore genetic loci, identify candidate genes, and uncover the regulatory mechanisms behind gene expression and phenotype formation. A case study involving the identification of candidate genes within the ARF transcription factor family demonstrates the platform's potential to aid in gene mining and functional genomics.

Compared with existing orchid databases, OrchidMD offers several significant advantages: (1) it consolidates and refines data from Orchidstra 2.0 and OrchidBase 6.0, resulting in a substantial enhancement in both data scale and platform responsiveness. (2) OrchidMD comprises the most extensive multi‐omics datasets thus far, encompassing 22 genome assemblies, transcriptomic data from 767 RNA‐seq libraries, 84 phenotypic traits, 6114 metabolites, and 26 proteome datasets. (3) It is evident that OrchidMD incorporates a number of pivotal tools, including GWAS, eFP Graph, Breed Recognition, CRISPR Design, and sRNA Target Prediction. The purpose of these tools is to facilitate the identification of genetic loci, candidate genes, and genetic variations, thereby providing a valuable set of resources for the field of orchid breeding. (4) The platform provides a plethora of common multi‐omics data analysis tools, applicable to all included orchid genomes. Consequently, OrchidMD serves as an efficient and accessible analysis hub (Table S6).

In addition, system performance testing with Apache JMeter (Nevedrov 2006) confirmed that OrchidMD supports multi‐user access with sub‐second median response times and stable concurrency handling. The underlying ThinkPHP + jQuery framework, combined with HTTPS encryption and role‐based access control, ensures that the platform remains both scalable and secure for long‐term use.

In summary, OrchidMD offers a valuable and versatile database for orchid breeding and functional genomics. With future updates incorporating new multi‐omics datasets and high‐efficiency methods, OrchidMD will continue to serve as an essential platform for advancing orchid research.

## 4 Methods

### 4.1 Data Sources

To build a comprehensive orchid multi‐omics database, we integrated data from genomics, transcriptomics, proteomics, phenotypic traits, and metabolomics (Figure 1; Tables S1–S4). The dataset includes 22 published orchid genome assemblies (17 from Epidendroideae species) and 569 981 genes (Table S1). Transcriptomic data from 767 RNA‐seq libraries were collected from various published studies and databases (Table S1). Phenotypic data covering 84 traits from 227 accessions were gathered (Table S2). The proteomics data are summarised in Table S3, and metabolomic data were retrieved from 20 previous studies (Table S4).

### 4.2 Reanalysis of Omics Datasets

To ensure consistency across datasets from different studies, we applied unified processing pipelines and parameters during data integration.

### 4.3 Genome Functional Annotation and Comparative Analysis

The 22 orchid genome assemblies were functionally annotated as described previously and integrated into the JBrowse of the genome synteny module (Buels et al. 2016). Transcription factors (TFs) in the 22 genomes were predicted using PlantTFDB v5.0 (Jin et al. 2016) and iTAK (Zheng et al. 2016). Amino acid sequences were uploaded to these databases for analysis. Gene families were identified based on homologous genes from Arabidopsis (TAIR; https://www.arabidopsis.org). Protein sequences were analysed using HMMER v3.2.1 (Potter et al. 2018) with an E‐value threshold of 1e‐5 to detect conserved motifs. Protein sequences from each genome pair were compared using Diamond v0.9.14.115 (Buchfink et al. 2015). Gene collinearity was identified using JCVI (MCScan) (Tang et al. 2024), and visualisations were generated using TBtools‐II v2.154 (Chen et al. 2023).

### 4.4 Ortholog Groups Among the Orchidaceae Genomes

The 569 981 genes from the 22 genome assemblies were clustered by first aligning protein sequences from each pair using mafft (Nakamura et al. 2018). Then, gene synteny was detected using MCScanX (Wang et al. 2012). T, and genes within syntenic regions were grouped into clusters. A total of 123 038 gene clusters were identified.

### 4.5 Transcriptome Analysis

RNA‐seq data were processed by clipping adapter sequences and removing low‐quality reads using fastp (v0.23.0) (Chen et al. 2018), Clean data were mapped to reference genomes using Hisat2 (v2.1.2) with default parameters (Kim et al. 2019). Gene expression levels were normalised using RSEM v1.3.3 with default settings in accordance with the same genome annotation (Li and Dewey 2011). Gene co‐expression networks were constructed by calculating Pearson correlation coefficients of gene expression pairs, retaining those with coefficients higher than 0.8.

### 4.6 GWAS

For GWAS, SNPs and indels with a minor allele frequency (MAF) lower than 0.05 were filtered out. The GWAS was conducted utilising GEMMA v0.98.1 (Zhou and Stephens 2012). Controlling for population structure with the first three principal components and an identity‐by‐state (IBS) kinship matrix. Significant associations were determined using the cutoff −log_10_(1/*n*), where *n* is the number of variations. Lead SNPs were identified by selecting the most significant variation within a locus.

### 4.7 GO and KEGG Enrichment Analysis

For GO and KEGG enrichment analysis, homologous gene pairs between each orchid genome and Arabidopsis were identified using blastp (Ye et al. 2006) Genes were annotated based on Arabidopsis GO and KEGG annotations, and libraries were created for each orchid genome. Enrichment analysis was performed using the “clusterProfiler” R package (Yu et al. 2012).

### 4.8 Architecture of the Breed Recognition Model

We conducted experiments using a fixed 6:2:2 split of the *Cymbidium* image corpus into training, validation, and test subsets; the validation subset was used solely for model selection, and final performance was assessed on the held‐out test subset (Goodfellow et al. 2016). The classifier employs a ResNet‐50 backbone. Each Bottleneck unit comprises a 1 × 1 convolution followed by batch normalisation and ReLU, a 3 × 3 convolution followed by batch normalisation and ReLU, and a final 1 × 1 convolution followed by batch normalisation without activation (He et al. 2016; Ioffe and Szegedy 2015). A squeeze‐and‐excitation module is inserted after this final batch normalisation and immediately before the residual identity addition, after which the block output is passed through ReLU (Hu et al. 2018). This placement applies channel re‐weighting to the transformed features prior to fusion with the skip path.

### 4.9 Transgenic Plants Constructs

The coding sequence (CDS) of the *CsiARF04* gene was amplified by PCR using gene‐specific primers and cloned into the transient overexpression vector pCAMBIA3302. The recombinant plasmid was then transformed into *Escherichia coli* DH5α competent cells. Positive clones were selected on kanamycin‐containing plates and verified by sequencing, resulting in the construction of the plant overexpression vector 35S::*CsiARF04*. The confirmed overexpression vector was introduced into *Agrobacterium tumefaciens* strain EHA105. A single colony was cultured overnight at 28°C and expanded until the optical density at 600 nm (OD₆₀₀) reached 1.0. The bacterial cells were harvested and resuspended in an infiltration buffer containing 100 mM MES, 100 mM MgCl₂, and 200 μM acetosyringone (AS). The suspension was adjusted to an OD₆₀₀ of 0.8 and incubated at room temperature for 6 h. Healthy rhizomes of *Cymbidium* species were selected and subjected to vacuum infiltration with *Agrobacterium suspensions* containing either the marker gene (3302‐Ruby), the empty vector (3302‐EV), or the recombinant 3302‐*CsiARF04* construct. Following infiltration, the rhizomes were incubated in the dark at room temperature for 2 days and then transferred to recovery and selection media for an additional 30 days. Successfully transformed rhizomes were confirmed by PCR (primers: F, 5′‐GCACGAGCCTACAGTTTAATT‐3′; R, 5′‐CTACTGATTGCATTCAGCATTATC‐3′) and by qRT‐PCR (primers: F, 5′‐TGCTGCGTGTGTGGATGGAA‐3′; R, 5′‐GCTGAGGATTGCGACTTGTCTG‐3′). The number of newly emerged buds in each treatment was subsequently recorded and subjected to statistical analysis.

### 4.10 Gene Editing Vector Construction and Editing Efficiency Detection

To construct the gene editing vector, the spCas9 coding sequence was inserted in place of the GUS gene within the pCAMBIA3301‐GUS backbone. Subsequently, a U6‐sgRNA expression cassette was cloned into the multiple cloning site (MCS). The resulting vector was transformed into *Agrobacterium tumefaciens* GV3101 competent cells. Positive single colonies were selected, inoculated into YEB liquid medium supplemented with rifampicin (50 μg/mL) and the relevant plasmid‐selective antibiotic, and cultured. Cells were harvested by centrifugation, resuspended in *Agrobacterium* infiltration buffer (10 mM MES, 10 mM MgCl₂, 200 μM acetosyringone [AS], 0.01% [v/v] Tween 20; pH 5.6), and adjusted to an OD₆₀₀ of 0.4 to prepare the infection solution. Young leaves of *C. sinense* were infiltrated using the agroinfiltration method. After 4 days of incubation, genomic DNA was isolated from infiltrated leaf tissue using the CTAB method (Steiner et al. 1995). The target region was PCR‐amplified using *CsiPDS*‐specific primers (forward: 5′‐GCACGAGCCTACAGTTTAATT‐3′; reverse: 5′‐CTACTGATTGCATTCAGCATTATC‐3′). PCR products were separated by agarose gel electrophoresis, and the expected bands were excised and gel‐purified. These purified amplicons were ligated into a TA‐compatible sequencing vector using the 5‐min TA/Blunt‐Zero Cloning Kit (Nanjing Vazyme Biotech Co. Ltd.; Cat# C601) as per the manufacturer's protocol. The ligation mix was then transformed into *Escherichia coli* DH5α competent cells. Finally, 20 randomly selected transformed single colonies were subjected to Sanger sequencing to determine gene editing efficiency.

### 4.11 Implementation

OrchidMD (http://www.orchidcomics.com) was constructed using the ThinkPHP (v5.0.24) framework and jQuery (v3.6.0) for the frontend, with Apache 2.4.53 as the web server and MySQL (v8.0.29) as the database engine. The database is accessible online without registration and optimised for use with Chrome, Opera, Firefox, Edge, and Safari on both Windows and macOS.

## Author Contributions

G.Z., F.Y., Q.L., and D.Y. designed and managed the project. Y.W., Z.L., Q.X. and W.L. performed all the coding of the website. J.L., J.G., Q.X., C.L., G.Y. and J.J. collected and processed data. Z.L. and C.L. tested the functions. F.Y., Q.L., Z.L., C.H., W.L. and Y.W. prepared the figures and wrote the manuscript. All authors read and approved the final manuscript.

## Conflicts of Interest

The authors declare no conflicts of interest.

## Supporting information


**Table S1:** Genome and transcriptome information of Orchidaceae species in OrchidMD.
**Table S2:**. Proteome information of Orchidaceae species in OrchidMD.
**Table S3:** Statistics of 84 phenotypic characters from 227 species of *Cymbidium sinense*.
**Table S4:** Metabolome information of Orchidaceae species in OrchidMD.
**Table S5:** Complete list of ARF transcription factors identified in the sixteen Orchidaceae genome.
**Table S6:** Comparison of OrchidMD, Orchidstra 2.0 and OrchidBase 6.0 platforms.

## Data Availability

Sources of all datasets are described in Supporting Information. All datasets are made available at http://www.orchidcomics.com.
